# Airway-resident memory CD4 T cell activation accelerates antigen presentation and T cell priming in draining lymph nodes

**DOI:** 10.1172/jci.insight.182615

**Published:** 2024-12-17

**Authors:** Caroline M. Finn, Kunal Dhume, Eugene Baffoe, Lauren A. Kimball, Tara M. Strutt, K. Kai McKinstry

**Affiliations:** Burnett School of Biomedical Sciences, Division of Immunity and Pathogenesis, College of Medicine, University of Central Florida, Orlando, Florida, USA.

**Keywords:** Immunology, Inflammation, Influenza, Memory, T cells

## Abstract

Specialized memory CD4 T cells that reside long-term within tissues are critical components of immunity at portals of pathogen entry. In the lung, such tissue-resident memory (Trm) cells are activated rapidly after infection and promote local inflammation to control pathogen levels before circulating T cells can respond. However, optimal clearance of Influenza A virus can require Trm and responses by other virus-specific T cells that reach the lung only several days after their activation in secondary lymphoid organs. Whether local CD4 Trm sentinel activity can affect the efficiency of T cell activation in secondary lymphoid organs is not clear. Here, we found that recognition of antigen by influenza-primed Trm in the airways promoted more rapid migration of highly activated antigen-bearing DC to the draining lymph nodes. This in turn accelerated the priming of naive T cells recognizing the same antigen, resulting in newly activated effector T cells reaching the lungs earlier than in mice not harboring Trm. Our findings, thus, reveal a circuit linking local and regional immunity whereby antigen recognition by Trm improves effector T cell recruitment to the site of infection though enhancing the efficiency of antigen presentation in the draining lymph node.

## Abstract 2

Beyond local protective impacts, airway-resident memory CD4 T cells speed antigen-bearing dendritic cell migration to draining lymph nodes resulting in faster naive T cell priming.

## Introduction

CD4 T cells can mediate strong protection against Influenza A virus (IAV) through multiple mechanisms, even in the absence of preexisting neutralizing Ab (1–5). Furthermore, T cells can recognize proteins that are highly conserved across IAV strains, unlike key neutralizing Ab targets that rapidly mutate. There is, thus, enthusiasm for harnessing memory CD4 T cells as a component of improved IAV vaccine strategies able to provide universal protection against diverse IAV strains (6–8). While some memory T cells circulate through the blood and lymphoid tissues, specialized tissue-resident memory T cells (Trm) reside long-term at sites of previous infection and play key roles in immunosurveillance (9). Indeed, lung Trm have been shown to contribute to optimal protection following challenges with diverse respiratory pathogens, including IAV (10–12). Virtually all studies centered on how Trm affect outcomes focus on their ability to rapidly modulate the tissue environments in which they reside. During IAV infection, CD4 Trm are activated by viral antigens within 40 hours of viral exposure, which substantially precedes the activation of T cells recognizing IAV-derived peptides in secondary lymphoid organs (13, 14). This rapid Trm response triggers a local inflammatory cascade that can control viral titers (13, 14) during what is otherwise the “stealth phase” of IAV infection (15), which precedes the vigorous activation innate immune defenses.

While Trm responses alone may be sufficient for T cell–mediated protection in some situations (16), IAV clearance has been shown in others to involve contributions from IAV-specific Trm and from other IAV-specific T cells that reach the lung several days after infection (17–19). The efficient priming of new antiviral effector T cells recognizing the virus requires transport of IAV antigens by migratory DC from the lungs to the mediastinal draining lymph nodes (dLN) (20). It is unclear whether the activation of lung Trm and of naive T cells responding to IAV antigens in the dLN are independent processes or if Trm activation has effects beyond those in the lung that affect the efficiency of T cell priming in secondary lymphoid organs. Understanding this relationship may lead to novel strategies for vaccines to better integrate local and regional T cell immunity against IAV and other respiratory pathogens.

Here, we investigated how recognition of antigen in the airways by IAV-primed lung CD4 Trm regulates presentation of the antigen, and the activation of naive T cells recognizing the antigen, in secondary lymphoid organs. To do so, we gave cognate fluorescently tagged protein, without additional adjuvant, i.n. to IAV-primed mice in order to recall a cohort of CD4 Trm with known antigen specificity. This approach allows for clear identification of the DC that take up the antigen in the lung (fluorescence^+^ DC), and for tracking antigen-bearing DC migration to secondary lymphoid organs (21). Importantly, this approach avoids triggering of pattern recognition receptors that are associated with infection that can independently promote DC activation (22) and pulmonary DC migration to secondary lymphoid organs (23). Furthermore, we analyzed effects of lung CD4 Trm activation on DC activation and migration in IAV-primed mice and in unprimed mice seeded with a physiological number of IAV-primed CD4 Trm through i.n. transfer. Comparing outcomes in these 2 models allows for determination of how changes in the cellular landscape of the lung and dLN that are induced by IAV infection facilitate or constrain the effects of CD4 Trm recall on antigen presentation dynamics. These changes include epigenetic modifications in populations of innate immune cells in the lung, including antigen presenting cells like alveolar macrophages (24); the formation of inducible bronchus-associated lymphoid tissue, which may provide important niches for Trm recall (25); and long-term changes in the cellularity of the dLN, which can affect T cell priming there (18, 26).

We show that antigen recognition by Trm in IAV-primed mice and by Trm in the airways of unprimed mice accelerates the activation and migration of antigen-bearing DC to the dLN. This improves the kinetics of priming of naive CD4 and CD8 T cells recognizing the i.n. administered antigen, which ultimately allows newly activated T effector cells to reach the lungs more quickly — an outcome that also requires CXCR3-dependent chemokine signals that are induced in the lung during Trm activation. Furthermore, we show that these mechanisms can have an adjuvant-like effect in promoting new T cell responses in the lungs in response to levels of antigen that are too low to have these effects in mice not harboring Trm. Finally, we show that airway CD4 Trm activation likewise accelerates T cell priming and the recruitment of newly activated T cells to the lungs during virulent IAV infection. These findings indicate that CD4 Trm activation has important consequences beyond the environment in which they respond that can dramatically improve T cell responses initiated in regional lymph nodes. Our results further underscore the benefits of targeting lung CD4 Trm generation through vaccination to protect against respiratory viruses.

## Results

### Airway and interstitial lung Trm are rapidly activated by airway antigen in IAV-primed mice.

**Figure 1:**
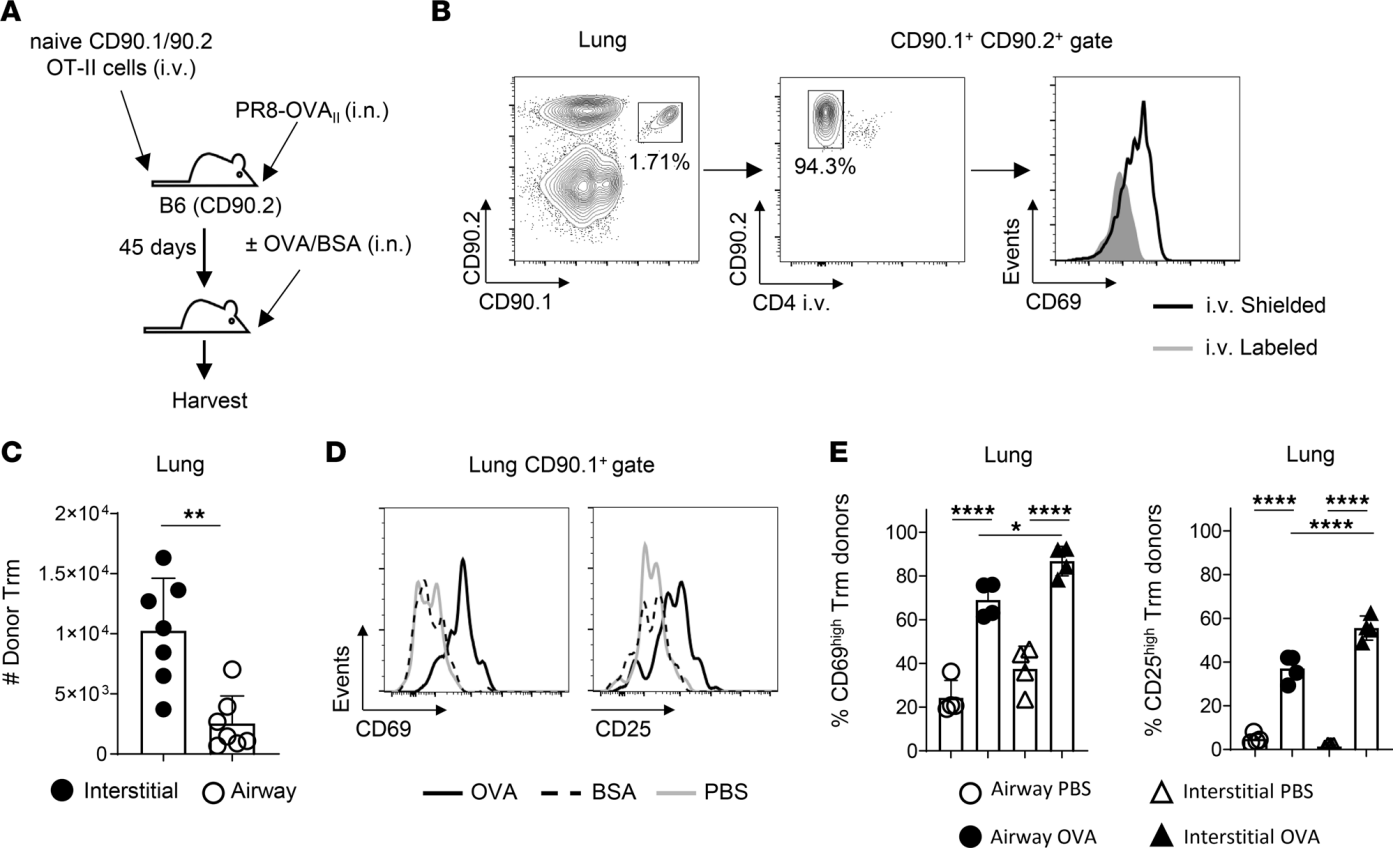
IAV-primed lung CD4 Trm respond rapidly to i.n. administered antigen. (**A**) B6 mice received 1 × 10^6^ naive CD90.1/CD90.2 OT-II cells i.v. followed by priming with PR8-OVA_II_. After 45 days, the primed mice were challenged with i.n. administered antigen to recall OT-II Trm in the lung. (**B**) Primed mice were treated at 45 dpi with fluorescent anti-CD4 Ab i.v. prior to lung harvest, with representative staining shown to identify donor OT-II cells (left), donor Trm shielding from labeling by i.v. administered CD4 Ab (center), and expression of CD69 by the i.v.^shielded^ versus i.v.^labeled^ OT-II cells (right). (**C**) Number of donor Trm (i.v.^shielded^) cells in interstitial and airway niches based on BAL harvest; *n* = 7/group; pooled from 2 experiments. (**D**) IAV-primed mice were given 50 μg of OVA or BSA, or PBS alone via i.n. administration. Representative CD69 (left) and CD25 (right) staining of total lung OT-II Trm 6 hours later. (**E**) The percentage of CD69^hi^ (left) and CD25^hi^ (right) donor Trm in airway (circles) and interstitial (triangle) niches from separate mice given OVA or PBS: *n* = 4–5 per group; results from 1 of 3 experiments. Student’s *t* test was used for pairwise comparison for **C**, and 1-way ANOVA with Tukey’s multiple-comparison test was used in **E**. *P < 0.05, **P < 0.01, ****P < 0.0001.

To investigate how antigen sensing by lung CD4 Trm affects antigen presentation in secondary lymphoid organs, we generated a Trm cohort of known T cell receptor (TCR) specificity as in our previous studies addressing requirements for lung CD4 Trm survival (27). We gave 1 × 10^6^ naive CD90.1^+^CD90.2^+^ OT-II TCR transgenic CD4 T cells i.v. to syngeneic CD90.2^+^ B6 hosts and then primed the mice with a sublethal i.n. dose of the mouse-adapted IAV strain PR8-OVA_II_ that expresses a peptide of ovalbumin (OVA) protein, which is recognized by the OT-II TCR (Figure 1A). The virus was delivered in a volume of 50 μL, which preferentially targets infection to the lungs versus the upper airways (28). At 45 days postinfection (dpi), most CD90.1^+^ donor OT-II cells detected in the lung fit canonical CD4 Trm criteria in that > 90% were shielded from labeling by fluorescent anti-CD4 Ab given i.v. shortly before lung harvest, indicating their location in a niche that is inaccessible to the vasculature (29). Furthermore, the i.v.^shielded^ population expressed higher levels of CD69, which is important for facilitating tissue retention by interfering with sphingosine-1-phospate–dependent signaling that promotes tissue egress (30) (Figure 1B). The CD69^hi^ i.v.^shielded^ phenotype is the most widely used discriminator of CD4 Trm versus circulating (CD69^lo^ i.v.^labeled^) memory subsets across murine studies (31).

Lung Trm can be divided based on location into those associated with the airways that are recoverable by bronchoalveolar lavage (BAL) and interstitial Trm that are not. About 25% of the i.v.^shieled^ OT-II Trm were recovered by BAL (Figure 1C). We performed phenotypic analysis to ask if the Trm recovered by BAL represent a fraction of a relatively homogenous lung Trm pool versus a subset with distinct attributes from interstitial Trm. Several markers that have been shown to discriminate Trm from circulating memory cells were differentially expressed by interstitial and airway Trm (Supplemental Figure 1; supplemental material available online with this article; https://doi.org/10.1172/jci.insight.182615DS1). These included lower CD127 and higher CD49a expression by airway versus interstitial Trm, both consistent with previous observations (32, 33). While interstitial Trm expressed less Ly6C than circulating memory cells, consistent with recent observations (34), we found Ly6C to be even further reduced on airway Trm. Levels of several adhesion molecules, including CD11a, CD49b, and CD49d, were also reduced on airway versus circulating Trm; differential expression of these molecules between airway and circulating TRM may be important in facilitating residency in this niche. Furthermore, airway Trm expressed less CD5 than interstitial Trm. CD5 expression by CD8 T cells may reflect sensitivity to IL-15 (35), suggesting that IL-15, which can sustain lung CD4 Trm survival (27), may differentially affect Trm in airway versus interstitial niches. Finally, while IFN-γ production after restimulation of both Trm subsets was similar, more interstitial Trm produced IL-2, TNF, IL-17, and IL-10 (Supplemental Figure 1). These results indicate striking phenotypic and functional heterogeneity between IAV-primed airway and interstitial Trm.

We next determined the kinetics of OT-II Trm recall by cognate antigen introduced into the lower airways by giving 50 μg of OVA protein i.n. in 50 μL of PBS. As controls, separate IAV-primed mice harboring Trm received either 50 μg of BSA, which is not recognized by the OT-II TCR, or PBS alone. We assessed Trm activation 6 hours after OVA administration, a time point at which DC have been shown to efficiently present MHC-II–restricted peptides derived from protein antigens delivered by the s.c. route (36). Despite their higher constitutive expression of CD69 versus circulating OT-II memory cells, most Trm further upregulated CD69, which is a commonly used early marker of T cell activation, and expressed higher levels of CD25, which is upregulated after CD69 following TCR triggering (Figure 1D). No signs of activation were induced by i.n. instillation of BSA or PBS alone (Figure 1D). When analyzed separately, 70% of airway and 90% of interstitial Trm upregulated CD69, with more interstitial than airway Trm also upregulating CD25 (Figure 1E). These results demonstrate rapid recall of the majority of interstitial and airway Trm in IAV-primed mice after cognate antigen delivery into the airways.

### CD4 Trm activation boosts numbers and activation of antigen-bearing DC in the dLN.

**Figure 2:**
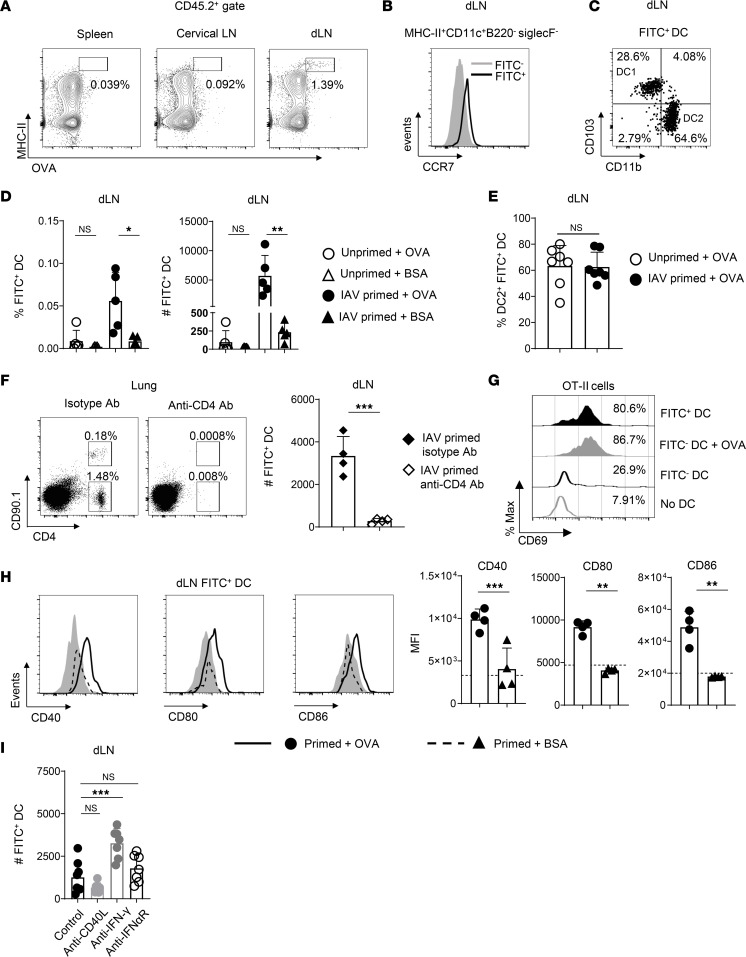
Lung CD4 Trm activation enhances the number and activation status of antigen-bearing DC in the dLN. (**A**) Representative staining of FITC^+^MHC-II^+^ cells in stated organs 20 hours after FITC-OVA administration to IAV-primed mice harboring OT-II Trm. (**B** and **C**) Representative CCR7 expression by FITC^+^ and FITC^–^ DC (**B**) and CD11b and CD103 expression by FITC^+^ DC to define DC1 and DC2 subsets (**C**). (**D**) frequency (left) and number (right) of FITC^+^ DC from stated mice 20 hours after antigen administration; *n* = 5/group; 1 of 2 experiments. (**E**) Frequency of DC2 within FITC^+^ DC of primed and unprimed mice receiving FITC-OVA; *n* = 7; results pooled from 2 experiments. (**F**) Representative staining of donor and host CD4 T cells in lungs of primed mice receiving FITC-OVA and isotype or CD4-depleting Ab treatment (left) and the number of FITC^+^ DC in the dLN (right); *n* = 4/group; 1 of 2 experiments. (**G**) CD69 expression by naive OT-II cells 48 hours after culture with stated subsets of sort-purified DC from IAV-primed mice 20 hours after FITC-OVA administration, with the percentage of CD69^+^ cells averaged from triplicate wells; 1 of 3 experiments. (**H**) Representative staining and MFI of CD40, CD80, and CD86 expression by FITC^+^ DC in dLN of primed mice after FITC-OVA or FITC-BSA administration, with shaded histograms and dotted lines in graphs for expression in mice receiving PBS; *n* = 4 per group; results from 1 of 3 experiments. (**I**) Numbers of FITC^+^ DC in dLN 20 hours after FITC-OVA administration to IAV-primed mice treated with stated Abs; *n* = 7/group; pooled results from 2 of 3 experiments. Student’s *t* test was used for pairwise comparison for all panels except **I**, where 1-way ANOVA with Dunnet’s multiple comparison test was used. *P < 0.05, **P < 0.01, ***P < 0.001.

To ask if antigen sensing by CD4 Trm can regulate presentation of peptides derived from the antigen in secondary lymphoid organs, we gave 50 μg of OVA protein labeled with FITC (FITC-OVA) or FITC-BSA to IAV-primed mice harboring OT-II Trm or to unprimed control mice. Monitoring the appearance of FITC^+^ cells in secondary lymphoid organs after i.n. administration of FITC-tagged antigen is a well-established approach to identify migratory antigen presenting cells (21). While no FITC^+^ cells were detected in the dLN 6 hours after FITC-OVA administration (not shown), FITC^+^ cells were detectable in the dLN by 20 hours (Figure 2A). In contrast, no FITC^+^ cells were detected at 20 hours in the spleen or in the cervical lymph nodes, which drain the upper airways (37) (Figure 2A). The FITC^+^ cells in the dLN at 20 hours expressed a CD45^+^MHC-II^+^CD11c^+^B220^–^SiglecF^–^ phenotype marking DC (Supplemental Figure 2). Furthermore, the FITC^+^ DC expressed higher levels of the chemokine receptor CCR7 than the FITC^–^ DC present in the dLN (Figure 2B), consistent with a migratory DC phenotype (38). About 70% of the FITC^+^ DC in the dLN were CD11b^+^CD103^–^, while the remainder were CD11b^–^CD103^+^ (Figure 2C). These phenotypes fit criteria marking conventional DC2 and DC1 subsets, respectively (39), and are consistent with the breakdown of FITC^+^ migratory DC subsets seen in similar models (23, 40).

The frequency and number of FITC^+^ DC detected in the dLN of unprimed mice given FITC-OVA or FITC-BSA, and in IAV-primed mice given FITC-BSA, were all similar (Figure 2D). Strikingly, about 10-fold more FITC^+^ DC were seen in IAV-primed mice given FITC-OVA (Figure 2D), but the proportion of FITC^+^ DC1 versus DC2 was similar in primed versus unprimed mice receiving FITC-OVA (Figure 2E). To confirm that the increase in migratory DC in the dLN of primed mice receiving FITC-OVA was due to the presence of OVA-specific CD4 Trm, we treated IAV-primed mice with CD4-depleting Ab prior to FITC-OVA administration to eliminate donor and any host OVA-specific memory CD4 cells generated by IAV priming. Thorough CD4 depletion in the lung via both i.p. and i.n. Ab administration (Figure 2F) reduced FITC^+^ DC detected in the dLN 20 hours after FITC-OVA administration to numbers similar to those in unprimed mice (Figure 2F). To verify that FITC^+^ cells were indeed presenting peptides derived from FITC-labeled antigen, we cultured naive OT-II cells in vitro with sort-purified FITC^+^ or FITC^–^ DC from dLN of mice given FITC-OVA. The OT-II cells were activated after 48 hours, as evidenced by CD69 upregulation, when cultured with FITC^+^ but not FITC^–^ DC, unless the FITC^–^ DC were pulsed with OVA peptide prior to culture (Figure 2G).

We next asked if antigen recognition by Trm affects qualitative properties of the migratory DC. Indeed, FITC^+^ DC in IAV-primed mice given FITC-OVA versus FITC-BSA expressed higher levels of the costimulatory molecules CD40, CD80, and CD86 twenty hours after antigen administration (Figure 2H), reflecting an enhanced activation status. The best characterized signals by which CD4 T cells affect APC activation are through CD40 ligand (CD40L) costimulation and IFN-γ production (41, 42). However, Ab treatment to block CD40L prior to FITC-OVA administration did not affect the number of FITC^+^ DC in the dLN, while, unexpectedly, IFN-γ–neutralizing Ab treatment increased FITC^+^ DC numbers (Figure 2I). Type I IFN has also been implicated in promoting DC activation (43, 44), but treatment with Ab to block the IFN-α/β receptor (IFNAR-1) did not affect detection of FITC^+^ DC in the dLN (Figure 2I). None of these treatments affected CD40, CD80, or CD86 expression by FITC^+^ DC or the proportion of DC1 versus DC2 (data not shown). These findings indicate that lung CD4 Trm activation accelerates antigen-bearing DC migration to the dLN and boosts their activation status, independently of major pathways governing CD4 T cell–dependent APC regulation in other settings.

### CD4 Trm activation accelerates antigen-bearing DC activation in the lung.

**Figure 3:**
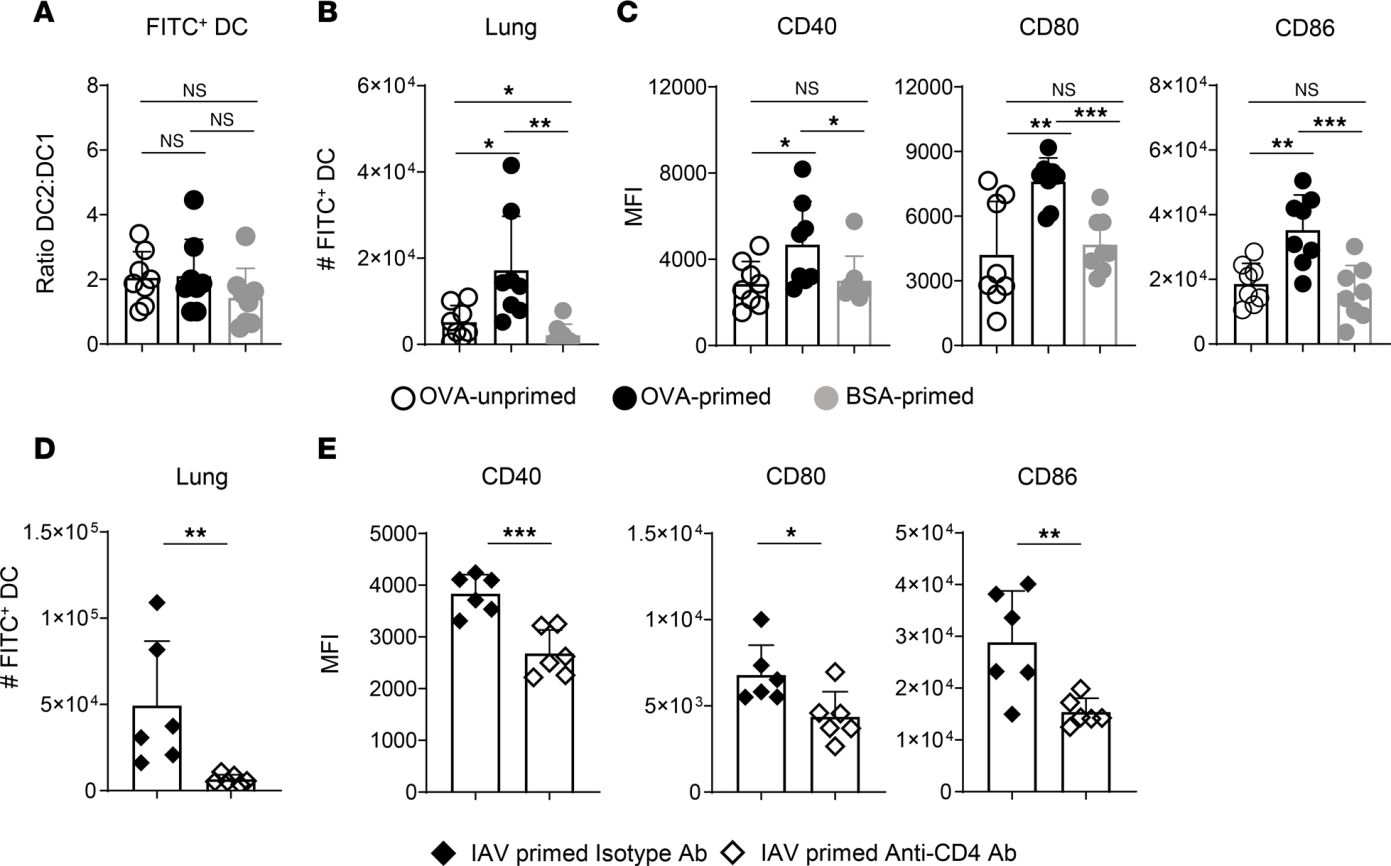
Rapid activation of lung DC presenting cognate antigen by CD4 Trm. IAV-primed mice harboring OT-II Trm or unprimed control mice were given 50 μg of FITC-OVA or FITC-BSA i.n. at 45 dpi. (**A**–**C**) The ratio of DC2/DC1 within FITC^+^ APC (**A**), the number of total FITC^+^ DC in the lungs of stated mice 6 hours after antigen administration (**B**), and MFI analysis of CD40, CD80, and CD86 expression by FITC^+^ lung DC in stated groups (**C**); *n* =8/group; pooled from 2 experiments. (**D** and **E**) The number of FITC^+^ lung DC and their expression of CD40, CD80, and CD86 6 hours after FITC-OVA administration to IAV-primed mice treated with isotype or CD4-depleting Ab; *n* = 6/group; results pooled from 2 experiments. One-way ANOVA with Tukey’s multiple-comparison test was used in **E** in **A**–**C**; Student’s *t* test was used for pairwise comparison in **D** and **E**. *P < 0.05, **P < 0.01, ***P < 0.001.

We next asked whether enhanced DC migration mediated by CD4 Trm activation correlates with more rapid activation of antigen-bearing DC in the lung. We thus assessed FITC^+^ DC in the lungs (Supplemental Figure 2) 6 hours after FITC-OVA administration to unprimed mice and in IAV-primed mice receiving FITC-OVA or FITC-BSA. This time point precedes FITC^+^ DC migration to the dLN, but TCR-dependent Trm activation is evident at 6 hours (Figure 1). While the proportion of DC1 versus DC2 within FITC^+^ DC was similar in all groups (Figure 3A), more FITC^+^ DC were detected in primed mice receiving FITC-OVA than in primed mice given FITC-BSA or in unprimed mice given FITC-OVA (Figure 3B). Furthermore, the FITC^+^ DC in lungs of primed mice receiving FITC-OVA expressed more CD40, CD80, and CD86 than in primed mice receiving FITC-BSA or in unprimed mice receiving FITC-OVA (Figure 3C). We also assessed FITC^+^ DC in lungs of primed mice 6 hours after receiving FITC-OVA that were depleted of CD4^+^ cells as in Figure 2. Depletion decreased the number of FITC^+^ DC (Figure 3D) and reduced their expression of CD40, CD80, and CD86 (Figure 3E). Antigen recognition by CD4 Trm, thus, increased the number and activation status of antigen-bearing DC in the lung within 6 hours of i.n. antigen exposure.

### Airway CD4 Trm activation in unprimed mice promotes enhanced antigen presentation in dLN.

**Figure 4:**
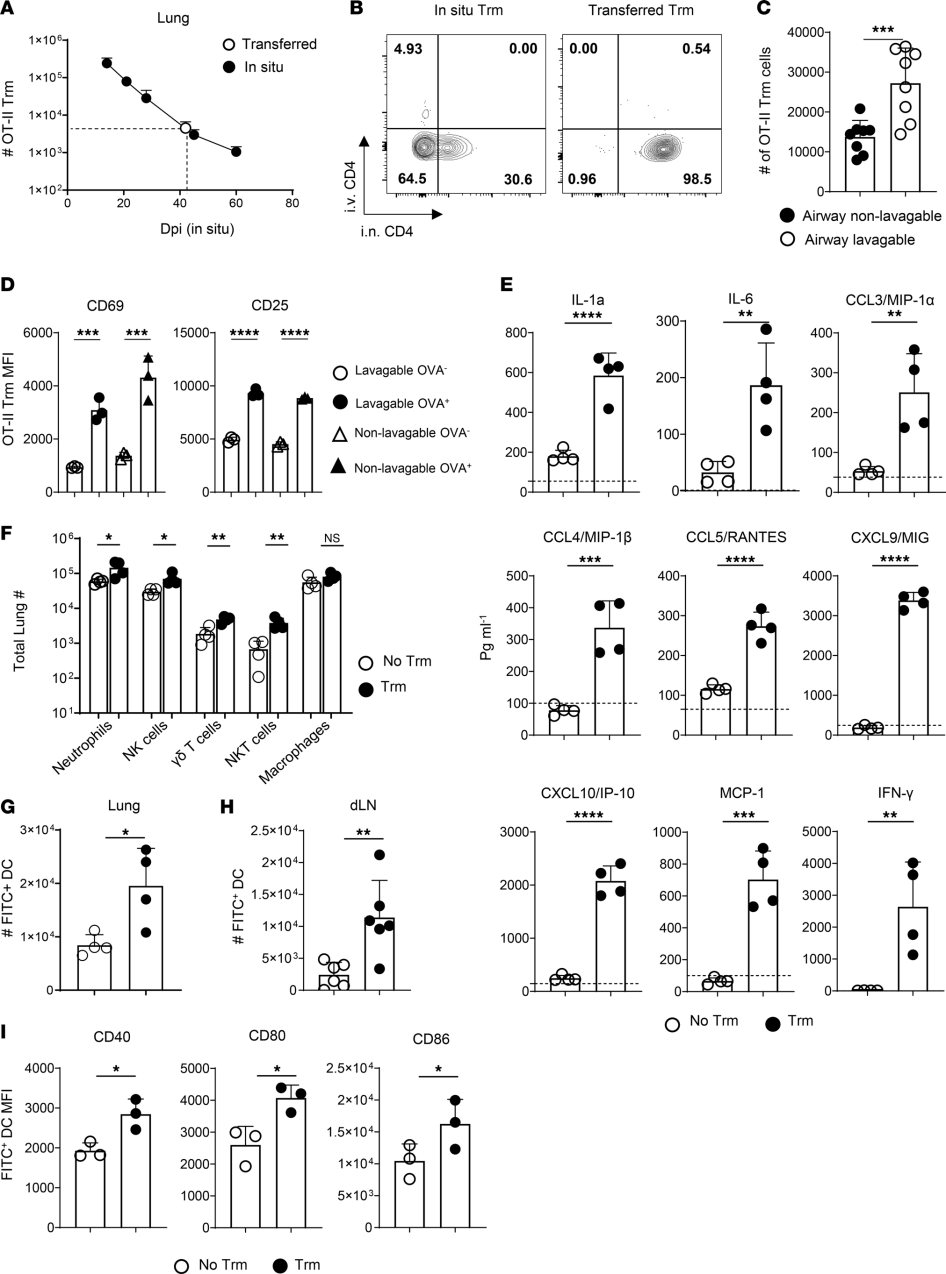
IAV-primed CD4 Trm rapidly respond to antigen in adoptive host mice after i.n. transfer. (**A**) The number of OT-II Trm in mice receiving naive OT-II cells i.v. on stated days after IAV priming (black) and the number of OT-II Trm in unprimed host mice 1 day after i.n. transfer of OT-II memory cells isolated from lungs of IAV-primed mice (open); *n* = 3 mice/group/time point. (**B** and **C**) Representative labeling of lung OT-II memory cells in primed mice at 45 dpi (left) and donor OT-II Trm in host mice 1 day after transfer (right) by noncompeting anti-CD4 Ab clones given i.n. and i.v. before lung harvest (**B**), and number of nonlavagable and lavagable donor Trm from unprimed host mice 1 day after transfer (**C**); *n* = 8/group; pooled from 2 experiments. (**D**) CD69 (left) and CD25 (right) expression by donor Trm subsets 6 hours after i.n. administration of FITC-OVA or PBS alone; *n* = 3–4/group; 1 of 3 experiments. (**E**) Levels of stated analytes in lung homogenates from mice receiving Trm or not 20 hours after FITC-OVA administration; dotted lines are average levels from mice receiving PBS alone; *n* = 4/group; 1 of 3 experiments. (**F**) Numbers of stated cell types in lungs of mice harboring Trm or not 20 hours after FITC-OVA administration; *n* = 4/group; 1 of 2 experiments. (**G** and **H**) Numbers of FITC^+^ DC in lungs (*n* =4/group; 1 of 2 experiments) (**G**) and dLN (*n* =6/group; pooled from 2 experiments) (**H**) 20 hours after FITC-OVA administration to unprimed mice harboring OT-II Trm or not. (**I**) CD40, CD80, and CD86 expression by FITC^+^ DC in the dLN; *n* = 3/group; 1 of 3 experiments. Student’s *t* test was used for pairwise comparison in all panels except **D**, where 1-way ANOVA with Tukey’s multiple-comparison test was used. *P < 0.05, **P < 0.01, ***P < 0.001, ****P < 0.0001.

IAV infection promotes structural changes and long-term alterations in the cellular landscapes of the lung (45–51) and dLN (18, 52). Aspects of these infection-driven changes could be required to facilitate Trm-dependent effects on antigen presentation in the dLN described above. We therefore asked the extent to which lung CD4 Trm can regulate lung DC migration to the dLN in unprimed mice. To do so, we isolated donor CD90.1^+^ memory OT-II cells from the lungs of IAV-primed B6 mice at 21–28 dpi and transferred 1 × 10^6^ i.n. to new unmanipulated CD90.2^+^ B6 host mice in a 50 μL volume to target the lungs. To verify that this approach seeds a physiologically relevant number of Trm, we compared the number of OT-II Trm detected in the lungs of adoptive hosts 1 day after i.n. transfer (Figure 4A) to numbers of Trm detected in the lungs of mice receiving naive OT-II cells prior to IAV priming as assessed from 14–60 dpi (Figure 4A). This analysis revealed a “take” of i.n. transferred Trm in unprimed host mice approximating the number of OT-II Trm derived from naive donor cells in the lungs of IAV-primed mice at 40 dpi.

We next compared the interstitial versus airway location of OT-II Trm in IAV-primed mice at 45 dpi and of the OT-II Trm in adoptive hosts 1 day after transfer using a FACS-based approach (4, 53). We gave 1 clone of anti-CD4 Ab i.v. and a noncompeting anti-CD4 Ab clone with a different fluorescent tag i.n. shortly before lung harvest. This analysis identified that the majority of Trm in IAV-primed mice were interstitial (i.n. Ab^shieled^/i.v. Ab^shielded^), with about 30% present in airway (i.n. Ab^labeled^/i.v. Ab^shielded^) niches (Figure 4B), closely matching proportions based on BAL recovery shown in Figure 1. In contrast, virtually all i.n. transferred Trm were associated with the airways (i.n. Ab^labeled^/i.v. Ab^shielded^) in adoptive hosts (Figure 4B). However, only about two-thirds of the transferred Trm could be recovered by BAL (Figure 4C), indicating that some Trm rapidly seed an airway-associated niche after i.n. transfer (evidenced by i.n. Ab labeling) that is inaccessible to recovery by BAL.

To ask if the transferred Trm remain localized to the lung, we first challenged mice receiving i.n. OT-II Trm with a sublethal dose of PR8-OVA_II_ 1 day after cell transfer and enumerated donor cells present in the lung, dLN, and spleen at 7 dpi. Infection drove modest Trm expansion in the lungs, but virtually no donor cells were detected in the dLN or spleen (Supplemental Figure 3), demonstrating locally restricted recall. We next assessed Trm activation in adoptive hosts in response to i.n. delivery of 50 μg of FITC-OVA. The Trm upregulated CD69 and CD25 within 6 hours, with similar patterns seen for Trm recovered by BAL or not recovered by BAL (Figure 4D), matching the rapid kinetics of in situ OT-II Trm recall shown in Figure 1.

CD4 Trm activation in IAV-primed mice rapidly induces a broad array of innate inflammatory mediators in the lung (13, 14). To test if antigen sensing by the transferred Trm could induce an inflammatory burst in unprimed mice, we assessed innate cytokines and chemokines in lung homogenates 20 hours after i.n. FITC-OVA administration to B6 mice harboring OT-II Trm or not. Levels of major inflammatory mediators, including IFN-γ, IL-6, CXCL9, CXCL10, CCL3, and CCL4, were at or near baseline in mice not harboring Trm, but all were strikingly upregulated in Trm recipients (Figure 4E). The inflammatory response in Trm recipients correlated with higher numbers of several innate immune cell subsets in the lungs, including neutrophils, NK cells, and γδT cells, that we previously showed to be activated by CD4 Trm responses against IAV (13, 27), as well as NKT cells, while alveolar macrophages were unchanged (Figure 4F). Representative staining to identify these innate immune subsets is shown in Supplemental Figure 4. Recognition of protein antigen by IAV-primed CD4 Trm in the airways of unprimed mice, thus, recapitulates hallmarks of their protective in situ response during IAV infection.

We next asked if antigen sensing by airway CD4 Trm in unprimed mice affects DC migration to the dLN. About 2-fold more FITC^+^ DC were detected in the lungs (Figure 4G) and about 5-fold more were detected in the dLN (Figure 4H) of Trm recipients 20 hours after FITC-OVA administration. Furthermore, the FITC^+^ DC in the dLN of Trm recipients expressed more CD40, CD80, and CD86 (Figure 4I). These results match the impacts of in situ Trm recall in IAV-primed mice summarized earlier and indicate that antigen sensing by airway CD4 Trm can enhance regional antigen presentation in the absence of any longer-term infection-induced changes in the lung or dLN.

### Trm-enhanced antigen presentation improves T cell priming efficiency in the dLN.

**Figure 5:**
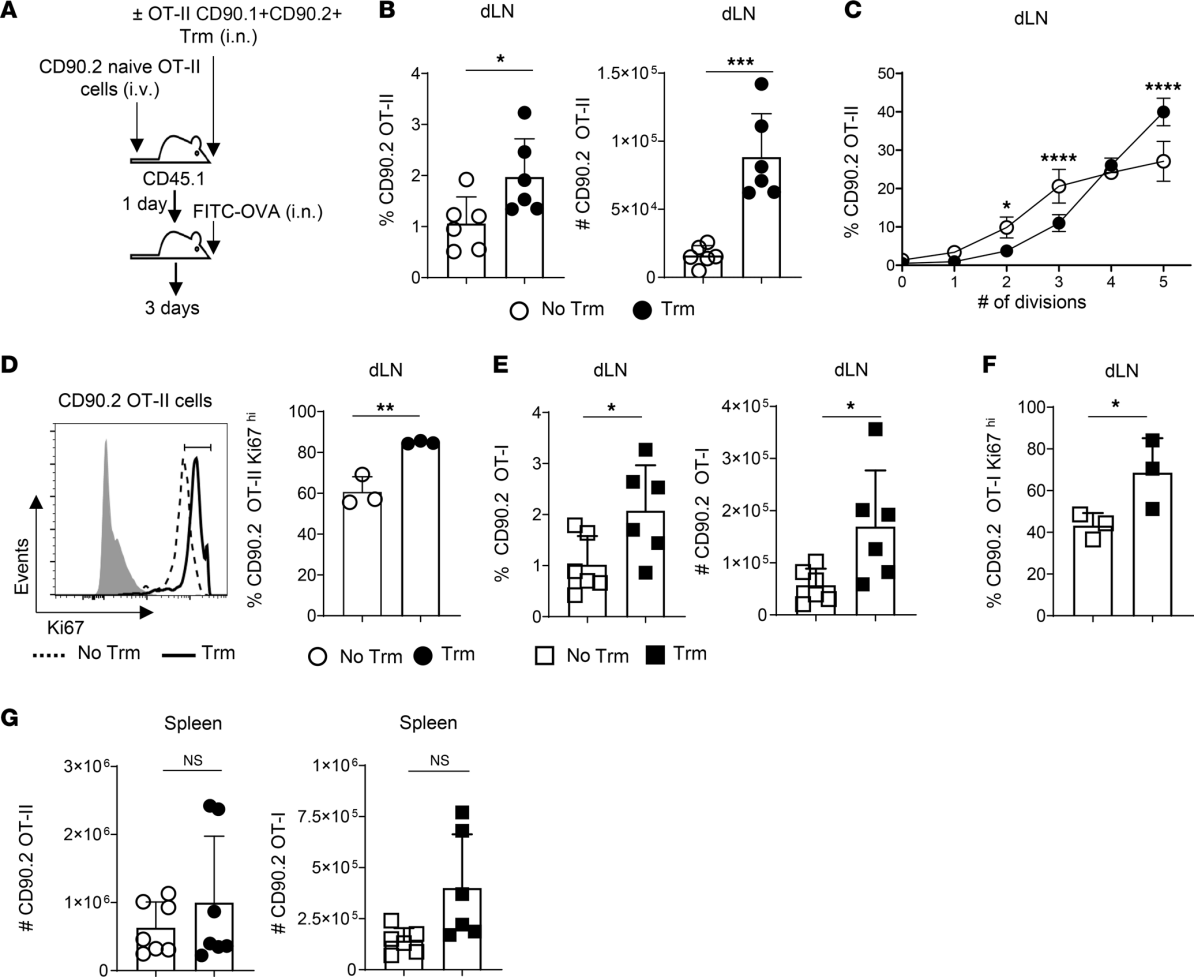
Airway Trm activation improves priming of naive T cells in dLN. (**A**) CD45.1^+^ B6 mice received 1 × 10^6^ naive CFSE-labeled CD90.2^+^ OT-II cells i.v. with or without i.n. transfer of 1 × 10^6^ CD90.1^+^CD90.2^+^ OT-II Trm. All mice were then challenged i.n. with 50 μg of FITC-OVA. (**B**–**D**) Frequency (left) and number (right) of cells derived from naive CD90.2^+^ OT-II donors in dLN after 3 days; *n* = 7; pooled from 2 experiments (**B**), with analysis of CFSE dilution (*n* = 4/group; 1 of 3 experiments) (**C**) and representative Ki-67 staining from naive OT-II responders (**D**), with bulk host T cells as shaded histogram (left), and the frequency of Ki-67^hi^ responders (right) from individual mice (*n* = 3/group; 1 of 3 experiments). (**E**) Frequency (left) and number (right) of responding cells derived from naive OT-I cells donor cells in the dLN of mice harboring OT-II Trm or not 3 days after FITC-OVA administration; *n* = 6/group; pooled from 2 experiments. (**F**) Frequency of Ki67^hi^ OT-I cells in dLN of mice harboring Trm or not; *n* = 3; 1 of 2 experiments. (**G**) Number of cells derived from naive OT-II (left) and OT-I (right) cells in the spleens of mice harboring Trm or not 3 days after i.n. FITC-OVA administration; *n* = 7/group for OT-II and 6/group for OT-I; results pooled from 2 experiments each with OT-II and OT-I cells. Student’s *t* test was used for pairwise comparison in all panels. *P < 0.05, **P < 0.01, ***P < 0.001.

We next asked if the boosted efficiency of antigen presentation in the dLN mediated by CD4 Trm activation can affect T cell priming. To do so, we transferred a cohort of naive CFSE-labeled CD45.2^+^CD90.2^+^ OT-II cells i.v. to CD45.1^+^ B6 hosts with or without i.n. transfer of IAV-primed CD90.1^+^CD90.2^+^ OT-II Trm. This approach allows for differentiation of host (CD45.1^+^), donor Trm (CD45.2^+^CD90.2^+^CD90.1^+^), and responders derived from naive donor cells (CD45.2^+^CD90.2^+^CD90.1^–^). One day later, we gave 50 μg of FITC-OVA in 50 μL of PBS i.n. to both groups of mice and analyzed the cells derived from naive donor cells in the dLN after 3 days (Figure 5A). About 4-fold more naive responders were detected in Trm recipients (Figure 5B). Furthermore, the cells derived from naive donors had divided more extensively based on loss of CFSE (Figure 5C) and expressed higher levels of the proliferation marker Ki67 (Figure 5D) than in mice not receiving Trm.

To ask if airway CD4 Trm activation can likewise enhance the kinetics of CD8 T cell priming, which requires cross-presentation in this model, we tracked responses of naive CD45.2^+^CD90.2^+^CD90.1^–^ donor OT-I TCR CD8 T cells in CD45.1^+^ B6 mice receiving i.n. transfer of CD45.2^+^CD90.2^+^CD90.1^+^ OT-II Trm or not. The OT-I TCR recognizes a peptide of OVA restricted to H-2K^b^ (54). More OT-I cells (Figure 5E) expressing higher levels of Ki67 (Figure 5F) were detected in mice harboring OT-II Trm. Lung DC can migrate from the dLN to the spleen after IAV infection to activate T cells there (55). However, while activated OT-I and OT-II cells were detected in the spleen, no differences in number were seen comparing mice harboring Trm or not (Figure 5G). Enhanced presentation of airway antigens in the dLN mediated by CD4 Trm activation, thus, promotes more rapid regional activation of naive CD4 and CD8 T cells.

### Airway Trm promote lung trafficking and enhanced functionality by newly activated T cells.

**Figure 6:**
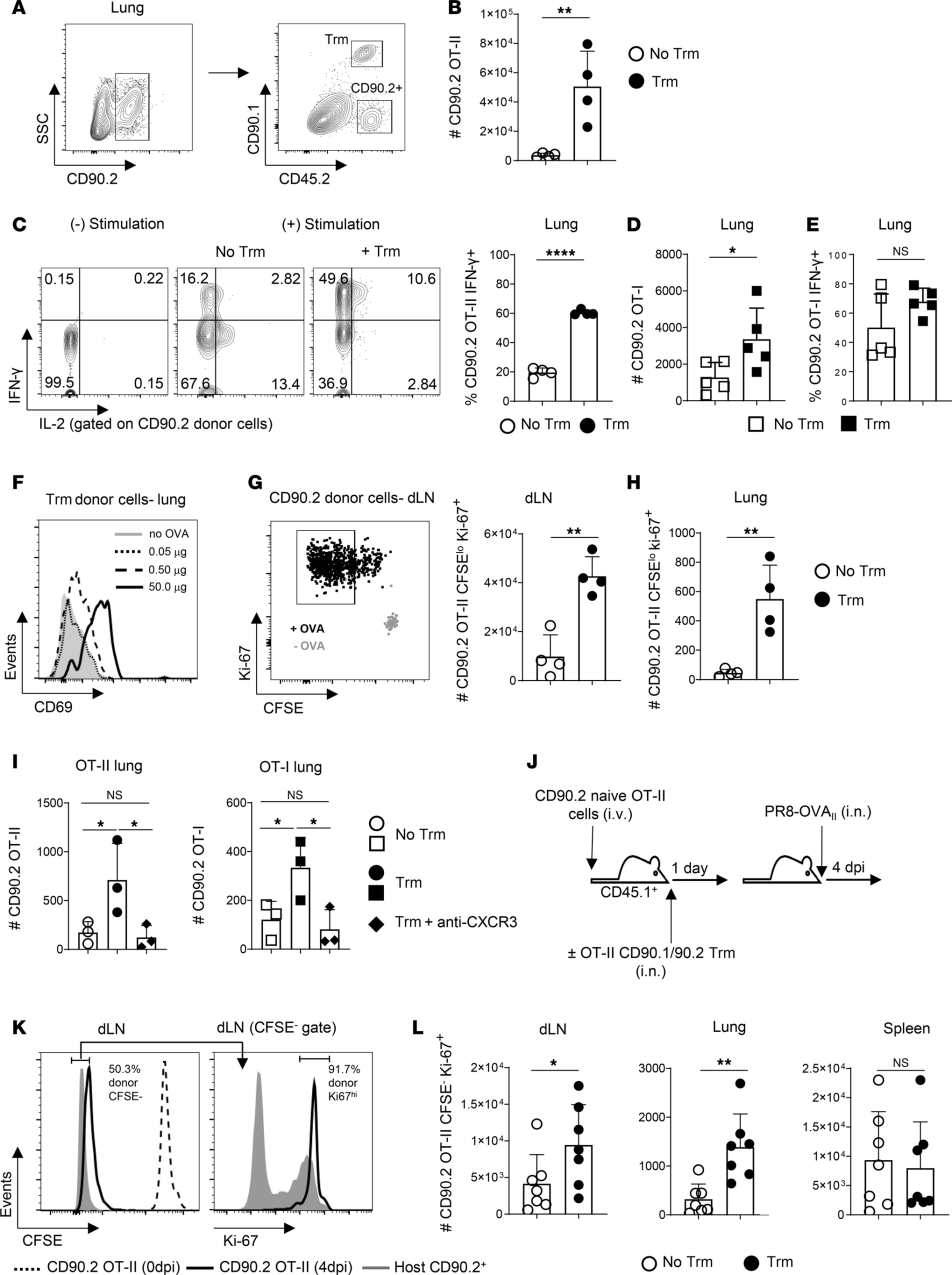
Airway CD4 Trm promote lung trafficking by newly activated T cells. CD45.1^+^ B6 mice receiving naive CD45.2^+^ CD90.2^+^ OT-II cells i.v. with or without CD45.2^+^CD90.1^+^CD90.2^+^ OT-II Trm i.n. were then challenged i.n. with 50 μg of FITC-OVA. (**A**) Representative staining to identify donor OT-II subsets. (**B**) Number of naive OT-II responders in lungs after 4 days. (**C**) Representative IFN-γ and IL-2 staining in mice without or without Trm, and frequencies of IFN-γ^+^ cells derived from naive donors; *n* = 4/group; 1 of 3 experiments. Separate mice received naive OT-I cells with or without OT-II Trm. (**D** and **E**) Number of OT-I responders in lungs (**D**) and IFN-γ**^+^** OT-I cells 4 days after FITC-OVA administration (**E**); *n* = 5/group; 1 of 2 experiments. (**F**) Representative CD69 expression by Trm 20 hours after i.n. challenge with 50, 0.5., or 0.05 μg of FITC-OVA. (**G** and **H**) Representative Ki67 and CFSE expression by naive OT-II responders in dLN of mice harboring OT-II Trm given 0.5 μg FITC-OVA or PBS (left) and numbers of CFSE^lo^Ki67^hi^ cells in dLN of mice harboring Trm or not at day 3 (right) (**G**), and in lungs at day 4 (**H**); *n* = 4/group; 1 of 3 experiments for each time point. Mice receiving naive donor cells and Trm were treated with CXCR3 blocking or control Ab prior to FITC-OVA administration. (**I**) Number of cells derived from naive OT-II (left) and OT-I (right) donors in lungs after 4 days; *n* = 3/group; 1 of 2 experiments. (**J**) Unprimed mice receiving CFSE-labeled naive OT-II cells with or without Trm were challenged with PR8-OVA_II_. (**K**) Gating to identify effector cells derived from naive donors. (**L**) Number of CFSE^–^Ki67^hi^ effectors at 4 dpi in stated organs; *n* = 7/group; pooled from 2 experiments. Student’s *t* test was used for pairwise comparison in all panels except **H**, where 1-way ANOVA with Tukey’s multiple-comparison test was used. *P < 0.05, **P < 0.01, ****P < 0.0001.

We next asked if Trm-dependent acceleration of naive T cell activation in the dLN affects responses by newly primed T cells in the lungs using the same adoptive transfer approach to separate transferred naive and Trm OT-II cells as described above (Figure 6A). While very few OT-II cells derived from naive donors were detected in the lungs 3 days after FITC-OVA administration in mice harboring Trm or not, about 10 times more were present in Trm recipients on day 4 (Figure 6B). To ensure that i.n. OVA challenge generated functional effector cells, we assessed the cytokine production capacity of newly activated OT-II cells present in the lungs. Markedly more newly primed OT-II cells produced IFN-γ in Trm recipients, with higher frequencies of IFN-γ/IL-2 double-positive cells, compared with newly primed cells in mice without Trm (Figure 6C). CD4 Trm recall also enhanced the number of OT-I effectors derived from naive donors in the lungs after FITC-OVA challenge, though not as dramatically (Figure 6D). Trm recall did not significantly affect IFN-γ production by OT-I effectors (Figure 6E), although a trend for more IFN-γ^+^ cells in mice harboring Trm was seen across experiments.

We next titrated the amount of FITC-OVA given i.n. to ask if airway CD4 Trm could promote new T cell responses in the lung against levels of antigen too low to stimulate lung infiltration otherwise. The Trm were activated by a 100-fold lower dose of FITC-OVA than used in the experiments described above as assessed by upregulation of CD69 at 6 hours, but not by 0.05 μg of FITC-OVA (Figure 6F). However, CD69 upregulation induced by 0.5 μg versus 50 μg of antigen was notably reduced. FITC^+^ DC were not reliably detectable in the lung or dLN after 0.5 μg FITC-OVA administration, but about 4-fold more activated (CFSE^lo^Ki-67^hi^) cells derived from naive OT-II cells were seen in dLN after 3 days in mice harboring Trm (Figure 6G). Moreover, while effectors derived from naive OT-II donors were virtually absent from lungs of mice not receiving Trm, they were readily detectable in Trm-bearing mice after 4 days (Figure 6H).

We reasoned that CD4 Trm–dependent chemokine signals may be required for the influx of new effectors into the lungs (Figure 6H). CXCR3-dependent chemokines promote T cell trafficking to the lung during IAV infection (56), and we detected rapid Trm-dependent induction of CXCL9 and CXCL10 in the lungs of unprimed mice receiving FITC-OVA (Figure 3). We therefore treated mice given Trm and naive OT-II or OT-I cells with CXCR3-blocking or control Ab prior to i.n. administration of 0.5 μg of OVA and enumerated effectors derived from the naive donor cells in the lungs 4 days later. CXCR3 blockade reduced numbers of newly activated CD4 and CD8 T cells to the very low numbers seen in mice not receiving Trm (Figure 6I). These results establish a regional circuit, linked through the activation of airway CD4 Trm_,_ that boosts the efficiency by which T cells recognizing airway antigens are primed in the dLN and the chemokine-dependent recruitment of newly activated effector T cells to the lung.

Finally, we asked if we could see Trm-dependent acceleration of naive T cell responses during IAV infection. To do so, we transferred naive CFSE-labeled CD90.2^+^ OT-II cells i.v., with or without i.n. transfer of IAV-primed CD90.2^+^CD90.1^+^ OT-II Trm, to unprimed CD45.1^+^ mice as in the experiments above, and we then infected the mice with PR8-OVA_II_ (Figure 6J). We enumerated CFSE^–^Ki67^hi^ responders derived from the naive donor cells at 4 dpi (Figure 6K), a time point at which antiviral CD4 T cell responses are not yet maximal in the dLN and when very few newly primed effector T cells are detected in the lungs during primary IAV infection. About 3 times more fully divided Ki67^hi^ effectors derived from naive donor cells were seen in the dLN and in the lungs of mice harboring Trm, but similar numbers were detected in the spleen (Figure 6L). These patterns match those seen assessing newly primed effector responses against FITC-OVA described above. Thus, even in the presence of infection-induced inflammation, airway CD4 Trm activation can accelerate regional T cell priming and the appearance of newly activated effector T cells in the lungs.

## Discussion

Virtually all investigation on how CD4 Trm affect immune responses focuses on their local effects in the tissues where they reside. For example, IAV-specific CD4 Trm activation by viral antigen initiates a rapid inflammatory burst in the lungs, resulting in the control of viral titers within 40 hours of infection (13, 14). While this response may be sufficient to clear IAV in some settings (16), optimal T cell–dependent IAV clearance in other studies has been shown to require Trm and virus-specific T cells that must be activated in secondary lymphoid organs before migrating to the lung (17, 18). We show here that, beyond coordinating local inflammatory environments, TCR-dependent CD4 Trm activation can accelerate naive T cell priming by promoting faster migration of highly activated antigen-bearing DC to the dLN, demonstrating another way in which the adaptive immune system can regulate innate immunity (57). We suggest that aspects of control by CD4 Trm on regional antigen presentation may be effective and targetable in a wide array of settings beyond IAV infection. For example, protective synergies between CD4 Trm and circulating T cells are described in mouse models using other pathogens including *Francisella tularensis* (58), *Chlamydia trachomatis* (59), and coronavirus (4). Linkages between tumor-resident and circulating T cells have also been described in models of cancer (60, 61). Negative effects have also been reported for synergies between local and systemic T cells in contributing to disease in models of vitiligo (62) and house dust mite allergen-induced asthma (63). Investigation to determine the extent to which Trm-mediated effects on regional antigen presentation influence the activation and effector functions of circulating T cells in these varied situations may reveal novel approaches to improve outcomes.

We found similar enhancements in the number and activation status of antigen-bearing DC in dLNs after delivery of cognate antigen to IAV-primed mice harboring OT-II Trm and to unprimed mice seeded with IAV-primed OT-II Trm. While Trm in IAV-primed mice could be divided into interstitial and airway compartments, all i.n. transferred Trm were associated with the airways of their adoptive hosts. We thus conclude that airway-associated CD4 Trm activation is sufficient to mediate the effects on regional antigen presentation that we report, which is teleologically appealing, since this location is on the frontline of antigen encounter. However, we further differentiated airway-associated Trm in adoptive hosts into lavagable (labeled by i.v. administered Ab and recoverable by BAL) and nonlavagable (labeled by i.v. administered Ab but not recoverable by BAL) compartments. Diverse leukocytes have recently been identified in lavagable and nonlavagable airway niches in IAV-primed mice using a similar approach (64). Further studies are required to determine how the precise location of airway CD4 Trm affects their interaction with different subsets of cells to promote the outcomes we describe, as this could provide important insight for strategies aimed at targeting Trm persistence in specific lung microenvironments to maximize their effects. Most of the cytokines and chemokines that we found upregulated in lung homogenates of Trm-bearing mice after i.n. FITC-OVA administration were also detected previously in supernatants of cultured lung CD4 Trm and cognate peptide-bearing DC (27). This supports the hypothesis that initial Trm-DC interactions serve as a “spark” to promote a broader lung inflammatory cascade (65). Here, we show that these same interactions also have consequences outside of the lung through promotion of enhanced presentation of airway antigens in the dLN.

We found both CD4 and CD8 T cell priming in the dLN to be accelerated after Trm recognition of airway antigens. This correlated with improved migration of antigen-bearing DC2 and DC1 from the lung to the dLN. These DC subsets are associated with preferential activation of CD4^+^ and CD8^+^ T cells, respectively (66). Unexpectedly, blockade of IFN-γ increased the number of migratory DC detected in the dLN of mice harboring OT-II Trm. Negative regulation of DC migration by IFN-γ has been seen in other settings, correlating with enhanced CCR5-dependent chemotaxis by the DC (67). Given that Trm activation boosted levels of IFN-γ and of CCR5 ligands (CCL3 and CCL4) in the lungs, we speculate that these signals may act to limit DC egress. Such regulation may be important to facilitate robust cognate interactions between antigen-bearing DC and effector T cells arriving in the lung during infection. Indeed, interactions with DC have been shown to be important for effector T cells in the lung to mediate antiviral activity (68) and can promote cytotoxic CD4 T cell differentiation during IAV infection (69). Notably, we found the number of FITC^+^ DC detected in the lung 6 hours after i.n. FITC-OVA administration was reduced following depletion of OT-II CD4 T cells. While it is possible that this outcome may in part be due to depletion of CD4^+^ DC (70), the fact that more FITC^+^ DC were also seen in in the lungs mice harboring OT-II Trm following i.n. delivery of FITC-OVA versus FITC-BSA suggests that cognate interactions with Trm may provide important survival signals to antigen-bearing DC. Indeed, MHC-II expression by DC has been found to support the survival of peptide-pulsed DC in vivo in other settings (71).

We found CD4 Trm activation to boost costimulatory molecule expression by antigen-bearing DC independently of IFN-γ and CD40L signals using Ab treatments based on previous experiments of ours in which these Ab clones were found to affect outcomes of IAV infection (72). Nevertheless, a limitation of the work presented here is that we cannot rule out the possibility that the Ab-mediated neutralization of these signals was incomplete. However, we previously showed that CD4 Trm–mediated control of IAV titers is independent of IFN-γ, CD40, and type I IFN signaling using KO mouse models (13). These results support the findings in this report that Trm-mediated control of DC migration is likewise independent of these signals. CD40L-independent DC activation by T cells has been reported by others (73) and may require strong TCR-mediated interactions (74). In contrast to our findings, IFN-γ was found to be critical for CD4 Trm–dependent protection and for optimal DC migration from infected lungs to dLN in a mouse model of coronavirus infection (4). These discrepancies indicate that key functional requirements may differ during CD4 Trm responses against different respiratory viruses. However, shared programming across infections may underlie the establishment and survival of lung CD4 Trm in airway and interstitial niches. This is supported by our findings that phenotypic markers distinguishing airway and interstitial Trm in IAV-primed mice, including CD11a, CD27, CD49d, CD127, and Ly6C, were also found to differentiate corona virus–specific CD4 Trm in these niches (4). Most Trm primed by viral infection fit Th1 criteria, but protective Th17-polarized IAV-specific Trm have been described (75), and dominant Th2 (76, 77) and Th17 (78, 79) lung Trm are seen in other settings. Whether such Trm can also accelerate regional antigen presentation, and if so, whether conserved or unique signals are involved across Th-polarization states, are important questions for future study.

We found that the number of IAV-primed lung CD4 Trm drop about 50-fold from 21 to 60 dpi, and similar rates of decline have been reported for IAV-primed CD8 Trm (80). Thus, even with optimal Trm priming through vaccination or infection, the time frame during which enough lung Trm are present to successfully combat a given dose of IAV without contributions from circulating T cells may be of limited duration. Indeed, we previously showed that a relatively high number, 3 × 10^6^, of i.n. transferred IAV-primed OT-II Trm could protect unprimed hosts against a lethal dose of PR8-OVA_II_ while 1 × 10^6^ Trm could not (27). Here, we show that i.n. transfer of 1 × 10^6^ IAV-primed OT-II Trm is, however, sufficient to accelerate regional antigen presentation and to establish chemokine gradients needed to recruit newly activated T cells to the lung. That a number of Trm beneath the threshold required to promote protection is nevertheless able to accelerate regional T cell responses is consistent with the concept that the mechanisms described likely grow in importance in situations where Trm recall must be augmented with antiviral T cells from systemic reservoirs. Indeed, newly primed antiviral T cells can be key contributors to IAV clearance even when high numbers of memory cells are responding as evidenced by experiments in which transferred IAV-specific memory CD4 T cells were shown to protect WT but not T cell–deficient hosts against the same IAV challenge dose (72).

Masopust and colleagues have shown that chemokine induction following CD8 Trm activation can recruit unstimulated memory T cells to sites of infection where they can be activated locally (81). Our finding demonstrating that CD4 Trm activation improves the kinetics of antigen delivery to the dLN, leading to faster T cell priming and the earlier appearance of new effector T cells in the lungs, reveal a separate way in which Trm can promote influx of protective T cells into tissues. Our preliminary studies also indicate that circulating memory T cell activation kinetics are similarly enhanced in the dLN following Trm activation in this model (our unpublished observations). Thus, incorporating CD4 Trm into IAV vaccine strategies may help to accelerate both primary T cell responses against newly encountered viral antigens while also promoting faster recall of circulating memory T cells in the dLN that target conserved viral epitopes. In this regard, investigating whether CD4 Trm in upper airways, another clinically relevant niche (82), can similarly affect regional T cell responses is important.

Finally, while our results show that antigen recognition by CD4 Trm can improve regional T cell priming during virulent IAV infection, we speculate that the CD4 Trm–dependent effects we describe may be especially effective in situations where APC activation is not triggered robustly though pattern-recognition receptors or when levels of antigen and inflammation are very low. Indeed, that CD4 Trm can promote lung responses by naive T cells against levels of antigen in the airways too low to do so otherwise indicates that CD4 Trm act as extremely sensitive amplifiers of low-end antigenic stimuli. This adjuvant-like effect of CD4 Trm may be highly beneficial in noninfectious settings — for example, in adoptive immunotherapy against cancers. Further study to test this possibility and to define the full scope of outcomes impacted by this mode of regulation may lead to new ways that CD4 Trm can be harnessed to prevent or manage disease in the respiratory tract and other tissue sites.

## Methods

### Sex as a biological variable.

Our study examined male and female animals, and similar findings are reported for both sexes.

### Mice.

WT C57BL/6J (B6) mice or CD45.1^+^ B6 mice (JaxBoy) were used as hosts in all experiments at 8–12 weeks of age. For adoptive transfer experiments, CD45.2^+^CD90^+^ OT-II mice, CD45.2^+^CD90.1^+^CD90.2^+^ OT-II mice, or CD45.2^+^CD90.2^+^ OT-I mice, all on a B6 background, were used at 4–8 weeks of age as a source of donor CD4 or CD8 T cells, respectively. The OT-II TCR recognizes aa 323–339 of OVA in the context of I-A^b^ (83), while the OT-I TCR recognizes aa 257–264 of OVA in the context of H-2K^b^ (54). All mice were originally obtained from The Jackson Laboratory, and all strains other than the OT-I mice were bred at the Lake Nona Vivarium at the University of Central Florida.

### Naive T cell isolation, adoptive transfers, and IAV infection.

Naive CD8^+^ or CD4^+^ cells from unmanipulated OT-I or OT-II mice, respectively, were obtained from pooled spleen and peripheral lymph nodes that were gently pressed through a nylon membrane to make a single-cell suspension. Single-cell suspensions were incubated on nylon wool (Polysciences) for 1 hour at 37°C to enrich for T cells followed by Percoll (MilliporeSigma) gradient separation to isolate small resting lymphocytes. Positive MACS selection using CD8 or CD4 microbeads (Miltenyi Biotec) was then performed to isolate OT-I or OT-II cells, respectively. The resulting cells were routinely > 95% CD8^+^ or CD4^+^ and expressed a naive phenotype (CD62L^hi^, CD44^lo^). In some experiments, donor cells were labeled with CFSE (Thermo Fisher Scientific) prior to injection into host mice in order to track cell division (84).

To generate OT-II Trm, CD90.2^+^ B6 mice received 1 × 10^6^ naive CD90.1^+^CD90.2^+^ OT-II cells in 200 μL of PBS by i.v. or retro-orbital (r.o.) injection. The mice were infected on the same day with an i.n. installation of a sublethal (0.2 LD_50_) dose of the mouse-adapted IAV strain PR8-OVA_II_ in 50 μL of PBS under isoflurane anesthesia. The same dose of PR8-OVA_II_ was used for experiments tracking responses OT-II cell responses against IAV. The viral stock was originally characterized at the Trudeau Institute and expresses the epitope of OVA recognized by the OT-II TCR in the hemagglutinin protein (85). All infected mice were monitored daily for infection-induced morbidity, including weight loss, hunched posture, ruffled fur, and reduced movement; mice were euthanized if humane endpoints were reached.

Donor CD90.1^+^CD90.2^+^ OT-II cells were reisolated from the lungs of PR8-OVA_II_–primed CD90.2^+^ B6 mice between 21 and 28 dpi as follows. Lung lobes were rinsed in PBS and then minced with a sterile razor blade and placed into C-tubes (Miltenyi Biotec) followed by digestion and homogenization using the mouse lung disassociation kit and a GentleMACS tissue dissociator with heaters (Miltenyi Biotec) as per the manufacter’s instructions. This was followed by isolation of live lymphocytes by Lympholyte gradient separation (Cedarlane). The cells were filtered extensively followed by positive selection of the donor cells using CD90.1 MACS beads. The number of donor cells was determined by flow cytometry, with purity generally > 85% and < 3% contaminating CD90.1^–^CD90.2^+^ cells. The OT-II Trm were transferred to new host mice under isoflurane anesthesia by i.n. instillation in 50 μL of PBS.

To track T cell responses against OVA, 5 × 10^5^ naive CD90.2^+^ CD4 or CD8 T cells obtained from OT-II or OT-I TCR transgenic mice, respectively, were transferred via r.o. injection to CD45.1^+^ B6 mice that also received Trm or not through i.n. transfer 1 day prior to OVA challenge.

### OVA administration and in vivo Ab treatment.

OVA, BSA, FITC-OVA, or FITC-BSA protein (Thermo Fisher Scientific) was administered to mice by i.n. instillation in 50 μL of PBS under isoflurane anesthesia. All antigen stocks contained < 0.03 endotoxin units/mL, as determined by Pierce gel clot endotoxin assay (Thermo Fisher Scientific). In some experiments, mice were treated with anti-CD40L (clone MR-1; BioXCell), anti–IFN-γ (XMG1.2; BioXCell), anti–type I IFN receptor (MAR1-583; BioXCell), or isotype control Ab via i.p. injection of 250 μg in 200 μL of PBS 1 day prior to OVA administration, and 100 μg of Ab was administered i.n. together with OVA. In other experiments, mice were treated with 250 μg of anti-CD4 Ab (GK1.5; BioXCell) i.p. 1 day prior to OVA administration and 100 μg was delivered i.n. together with OVA administration.

In some experiments, anesthetized IAV-primed mice were injected i.v. with 3 μg of APC-labeled anti-CD4 Ab (GK1.5, BioLegend) in 100 μL of PBS with or without 1.5 μg of PE-labeled anti-CD4 (RM4.4, BioLegend) i.n. in 50 μL of PBS prior to euthanasia and organ harvest. GK1.5 and RM4.4 are noncompeting clones in terms of binding CD4.

### Tissue collection.

All experimental mice were euthanized by cervical dislocation. For assessment of responses to airway antigens, exsanguination was performed by perforation of the abdominal aorta and lung perfusion by injection of PBS through the right ventricle of the heart. Lungs, spleen, and lymph nodes were harvested into RMPI 1640 media (Thermo Fisher Scientific) supplemented with 7.5% fetal bovine serum (Hyclone), 2 mM L-glutamine (Thermo Fisher Scientific), 100 IU penicillin and 100 μg/mL streptomycin (Thermo Fisher Scientific), 10 mM HEPES (Thermo Fisher Scientific), and 50 μM 2-mercaptoethanol (MilliporeSigma). Lymph nodes were prepared into single-cell suspensions using an optimized mechanical disruption protocol by gentle and thorough pressing through a taut stainless-steel mesh (80 μM) using a sterile rubber plunger from a 5 mL syringe. The mesh and plunger were then rinsed with 3 mL of media. This procedure was repeated 3 times. Spleen and lung were processed by enzymatic digestion using a gentleMACS dissociator and enzyme kits specific for the lung or for spleen (Miltenyi Biotec) to generate single-cell suspensions. All single-cell suspensions were filtered through a 70 μM nylon cell strainer (Thermo Fisher Scientific) prior to further analysis.

In some experiments BAL was collected by administering 1 mL of sterile PBS into the lungs via the exposed trachea and then gently retracting the fluid. This process was repeated 3 times per mouse, pooling the volume.

In some experiments CD45^+^MHC-II^+^B220-CD11c^+^ cells were sort purified from the dLN of mice 1 day after i.n. FITC-OVA administration using a CytoFlex SRT sorter (Beckman Coulter) into FITC^+^ and FITC^–^ populations. Some FITC^–^ cells were pulsed with 10 μg/mL of OT-II peptide directly after sorting. All sorted APC populations were cocultured 1:1 with naive OT-II cells for 48 hours prior to analysis of OT-II cells for CD69 expression by flow cytometry.

### Flow cytometry.

Single-cell suspensions were washed, resuspended in FACS buffer (PBS plus 0.5% BSA and 0.02% sodium azide), and incubated on ice with 1 μg of CD16/CD32 blocking Ab (2.4G2; BioXCell) and optimized concentrations of the following fluorochrome-labeled Abs for surface staining. The following were from BioLegend, anti-CD45.2 (104, PE-Cy7), anti-CD49a (HMα1, APC), anti–Ly-6C (HK1.4, FITC), anti-CD25 (PC61, PE), anti–MHC-II (M5/114.15.2, PerCP), anti-CD11c (N418, efluor-450), anti-SiglecF (E50-2440, APC-Cy7), anti-CD64 (S18017D, APC), anti-CD40 (3/23, APC), anti-CD80 (16-10A, PE), anti-CD86 (GL-1, PE), anti-NK1.1 (PK136, PE). The following were from BD Biosciences, anti-CD11a (2D7, FITC), anti-CD49d (9C10, PE), anti-CD44 (IM7, PE), and anti-B220 (RA3-6B2, V500). Anti-CXCR6 (221002, PE) was purchased from R&D. The following were from Thermo Fisher Scientific, anti-CD90.1 (HIS51, PerCP), anti-CD90.2 (53-2.1, efluor-450), anti-CD4 (RM4.5, PE), anti-CD8 (53-6.7, PE), anti-CD127 (sb/199, FITC), anti-CD27 (A7R34, APC), anti-CXCR3 (CXCR3-173, FITC), anti-CD69 (H1.2F3, PE), anti-CD62L (MEL-14, APC), anti-CCR7 (4B12, PE), anti-CD103 (2E7, PE), anti–GR-1 (RB6-8C5, PE), anti-γδTCR (eBioGL3, APC), anti-CD3 (17A2, PerCP), and anti-CD11b (M1/70, APC). For intracellular cytokine staining, cells were stimulated for 4 hours with 10 ng/mL PMA (MilliporeSigma) and 50 ng/mL ionomycin (MilliporeSigma), and with 10 mg/mL brefeldin A (MilliporeSigma) added after 2 hours. Cells were then surface stained and fixed for 20 minutes in 4% paraformaldehyde followed by permeabilization for 10 minutes in 0.1% saponin buffer (PBS plus 1% FBS, 0.1% NaN_3_, and 0.1% saponin). The cells were then stained for cytokine by the addition of fluorescently labeled anti–IFN-γ (XMG1.2; Thermo Fisher Scientific) and anti–IL-2 (JES6- 5H4; Thermo Fisher Scientific) Abs for 20 minutes. Detection of Ki67 (SolA15, Thermo Fisher Scientific) was conducted using the Foxp3 staining buffer set (Thermo Fisher Scientific). Zombie NIR Fixable Viability kit (BioLegend) was used to discriminate live from dead cells.

All FACS analysis was performed using BD FACSCanto II (BD Biosciences) or Cytoflex (Beckman Coulter) flow cytometers and FlowJo (BD Biosciences) analysis software.

### Detection of pulmonary cytokines and chemokines.

Levels of cytokines and chemokines in lung homogenates collected as described previously (13) were determined using mouse multiplex kits (MilliporeSigma) read on a Bio-Plex Multiplex 200 Luminex reader (Bio-Rad).

### Statistics.

Unpaired, 2-tailed Student’s *t* tests were used to assess whether the means of 2 normally distributed groups differed significantly. The Welch correction was applied when variances were found to differ. One-way ANOVA with appropriate multiple-comparison test was used to compare multiple means. *P* < 0.05 was considered significant. Data are shown as mean ± SD. Statistical analysis was performed using GraphPad Prism software (GraphPad Software).

### Study approval.

All experimental animal procedures were approved by and conducted in accordance with the University of Central Florida’s Animal Care and Use Committee’s guidelines under protocol no. 202300114.

### Data availability.

Values for all data points in graphs are reported in the Supporting Data Values file.

## Author contributions

CMF and KKM contributed to conception, experimental design, analysis, and interpretation of data and prepared the manuscript. CMF, EB, LAK, and KD contributed to data acquisition. TMS contributed to data acquisition, interpretation of data, and manuscript preparation.

## Supplementary Material

Supplemental data

Supporting data values

## Version 1 12/17/2024

In-Press Preview

## Version 2 02/10/2025

Electronic publication
