# Supplementary material for: Airway-resident memory CD4 T cell activation accelerates antigen presentation and T cell priming in draining lymph nodes
Source: JCI Insight. 2024 Dec 17;10(3):e182615. doi: 10.1172/jci.insight.182615 (PMC11948587; doi:10.1172/jci.insight.182615)
Supplement: Supplemental data [file jciinsight-10-182615-s119.pdf]

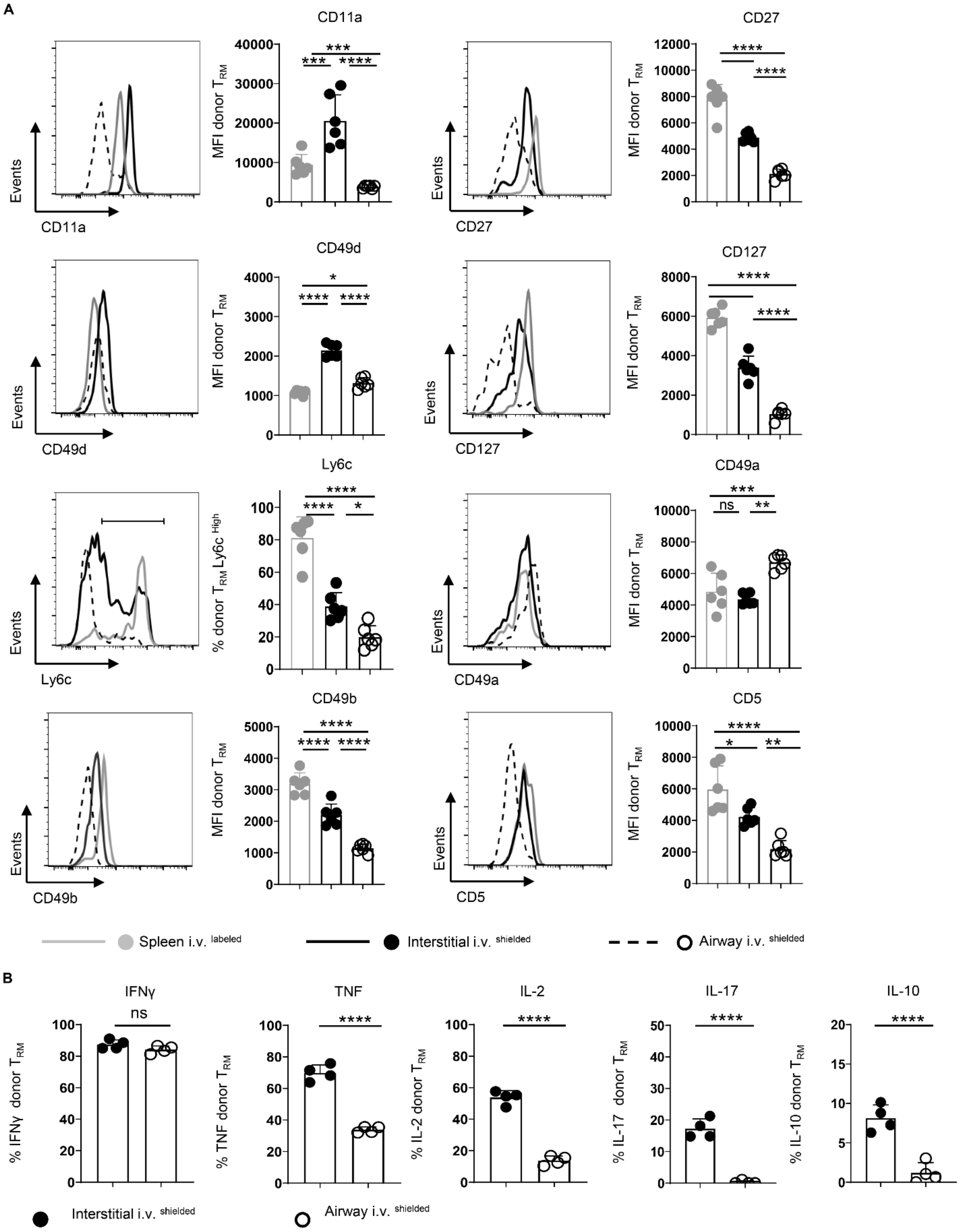

A
CD11a
MFI donor T_{RM}
Events
CD11a
CD27
CD49d
CD127
Ly6c
% donor T_{RM} Ly6c ^{High}
CD49a
CD49b
CD5
Spleen i.v. labeled
Interstitial i.v. shielded
Airway i.v. shielded
B
IFNγ
TNF
IL-2
IL-17
IL-10
% IFNγ donor T_{RM}
% TNF donor T_{RM}
% IL-2 donor T_{RM}
% IL-17 donor T_{RM}
% IL-10 donor T_{RM}
ns
Interstitial i.v. shielded
Airway i.v. shielded

Supplemental Figure 1: *Airway and Interstitial T_{RM} are characterized by unique phenotypes and functional potential.* **(A)** Representative staining and summary analysis for stated marker expression by OT-II memory cells in IAV-primed mice at 45 dpi by splenic memory cells labeled with anti-CD4 antibody shortly before organ harvest (i.v.^{labeled}), i.v.^{shieled} cells from the lungs not recoverable by BAL, and i.v.^{shielded} cells recovered by BAL. n=6/group; pooled from 2 individual experiments. **(B)** The frequency of interstitial and airway memory OT-II cells positive for stated cytokines after restimulation; n=4/group; 1 of 3 experiments. One-way ANOVA with Tukey's post-test used in (A) and Students t test used in (B).

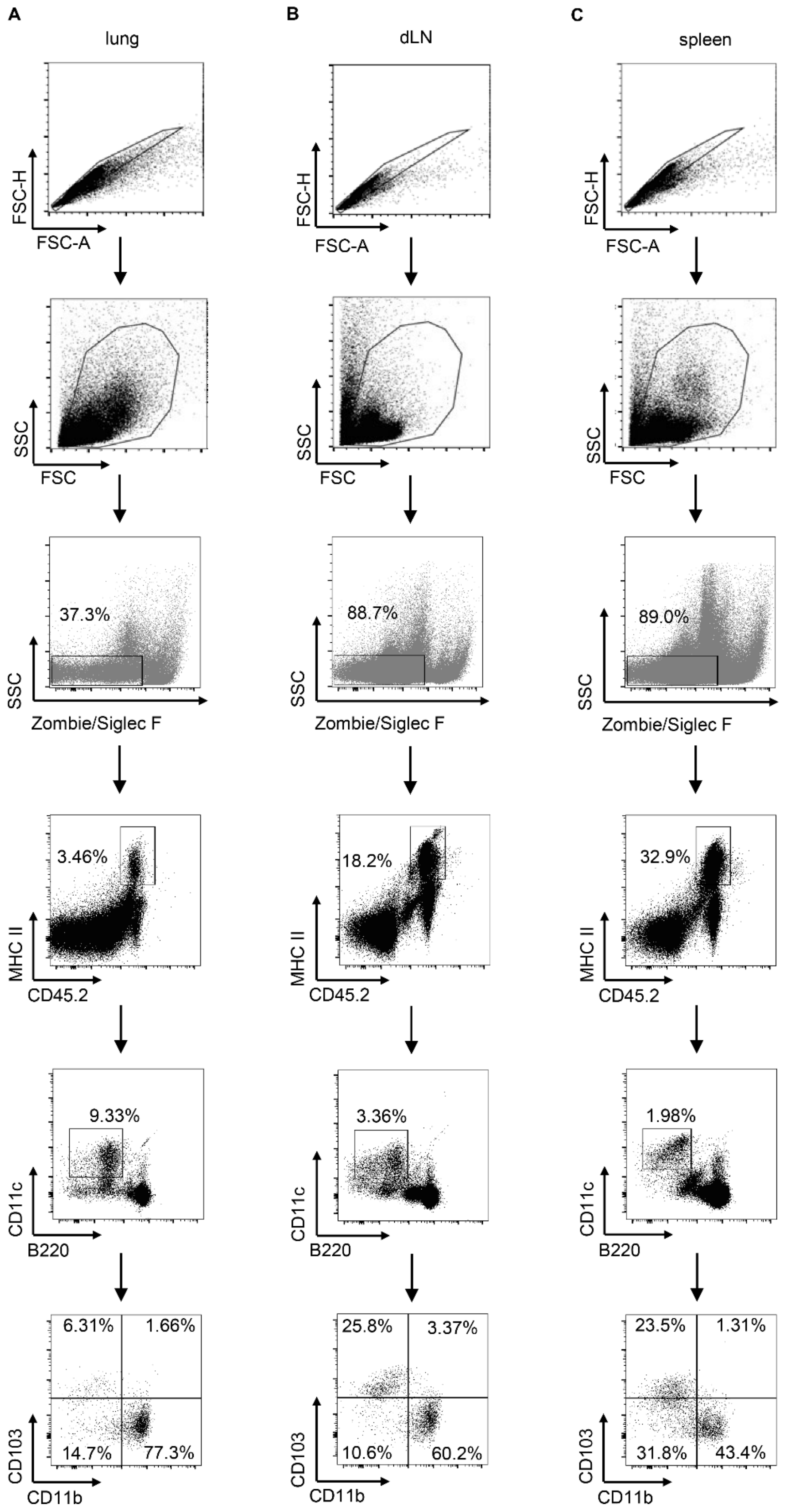

Supplemental Figure 2: *Representative staining* of *gating strategies for FACs identification of DC in lung (**A**), dLN (**B**) and spleen (**C**)*. Mice were primed with PR8-OVA_{II} and challenged 45 dpi i.n. with 50 ug of FITC-OVA. Organs were harvested at 1 day post OVA installation.

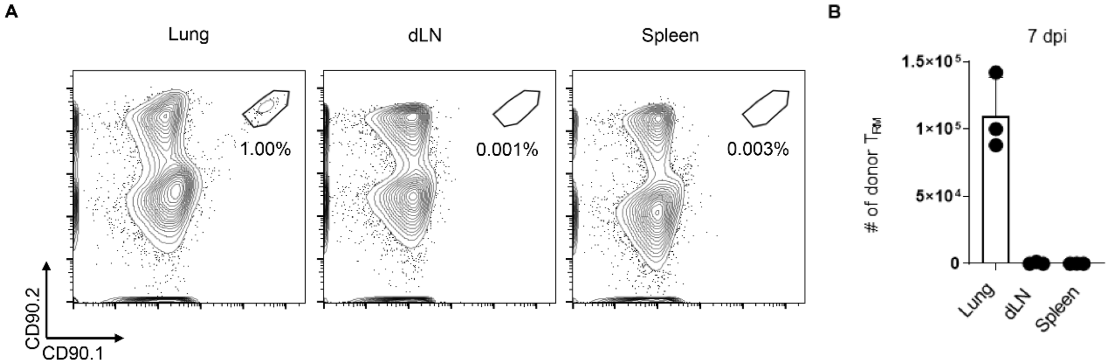

Supplemental Figure 3: *IAV-primed CD4 T_{RM} respond locally against IAV in adoptive hosts.* CD90.2$^+$CD90.1$^+$ OT-II memory cells were isolated from the lungs of PR8-OVA_{II}-primed mice at 26 dpi and 1x10^6 were transferred i.n. to new unprimed B6 mice. The mice were then challenged with a sublethal dose of PR8-OVA_{II}. Shown is representative staining to identify donor memory-derived cells at 7 dpi in stated organs **(A)** and the number of donor cells detected in individual mice **(B)**; n = 3 mice; results from 1 of 2 experiments.

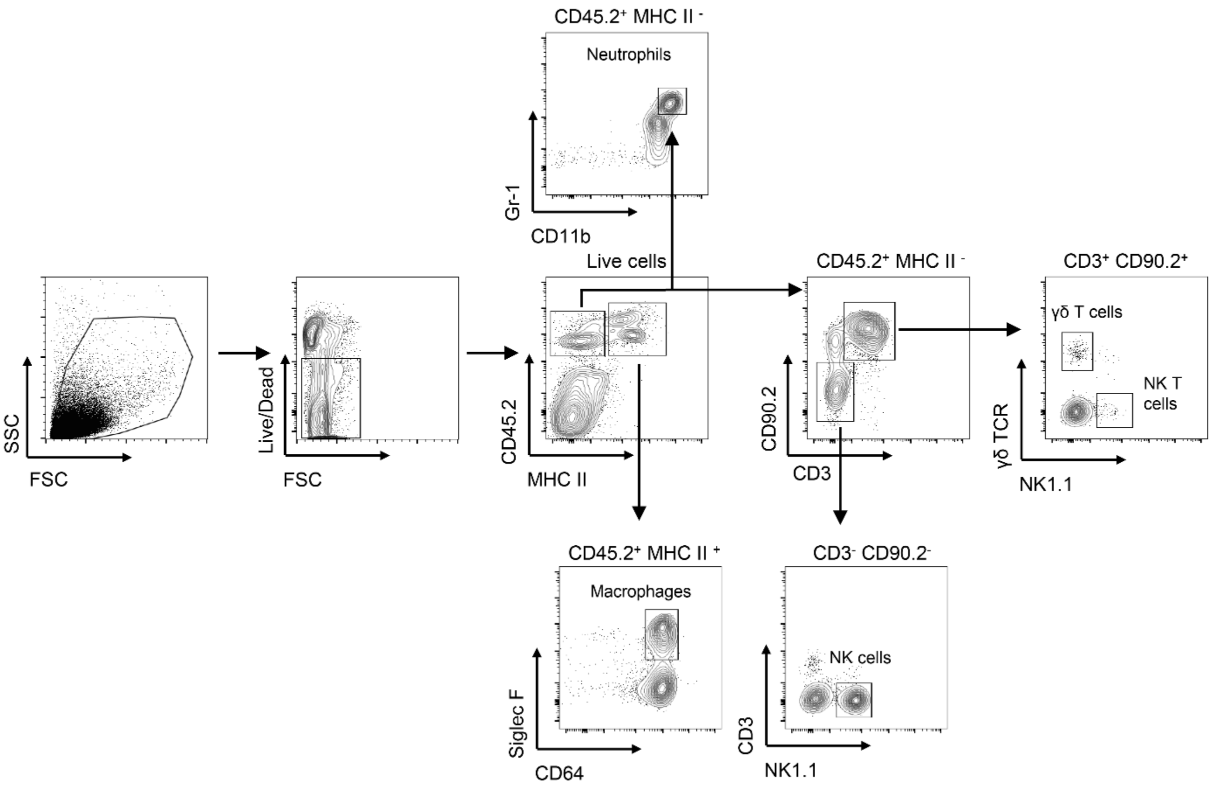

Supplemental Figure 4: *Representative staining of gating strategies used to identify innate cells populations in the lungs summarized in Figure 4F.*
